# Chemical‐Driven Outflow of Dissociated Amyloid Burden from Brain to Blood

**DOI:** 10.1002/advs.202104542

**Published:** 2022-02-02

**Authors:** Donghee Lee, Hyunjin Vincent Kim, Hye Yun Kim, YoungSoo Kim

**Affiliations:** ^1^ Department of Pharmacy College of Pharmacy Yonsei University 85 Songdogwahak‐ro Yeonsu‐gu Incheon 21983 South Korea; ^2^ Yonsei Institute of Pharmaceutical Sciences College of Pharmacy Yonsei University 85 Songdogwahak‐ro Yeonsu‐gu Incheon 21983 South Korea; ^3^ Korea Institute of Science and Technology (KIST) University of Science and Technology (UST) 5 Hwarang‐ro 14‐gil Seongbuk‐gu Seoul 02792 South Korea; ^4^ Department of Integrative Biotechnology and Translational Medicine Yonsei University 85 Songdogwahak‐ro Yeonsu‐gu Incheon 21983 South Korea

**Keywords:** alzheimer's disease, Aβ disaggregation, Aβ plaques, blood diagnosis, plasma Aβ

## Abstract

Amyloid‐β (Aβ) deposition in the brain is a primary biomarker of Alzheimer's disease (AD) and Aβ measurement for AD diagnosis mostly depends on brain imaging and cerebrospinal fluid analyses. Blood Aβ can become a reliable surrogate biomarker if issues of low concentration for conventional laboratory instruments and uncertain correlation with brain Aβ are solved. Here, brain‐to‐blood efflux of Aβ is stimulated in AD transgenic mice by orally administrating a chemical that dissociates amyloid plaques and observing the subsequent increase of blood Aβ concentration. 5XFAD transgenic and wild‐type mice of varying ages and genders are prepared, and blood samples of each mouse are collected six times for 12 weeks; three weeks of no treatment and additional nine weeks of daily oral administration, ad libitum, of Aβ plaque‐dissociating chemical agent. By the dissociation of Aβ aggregates, the altered levels of plasma Aβ distinguish between transgenic and wild‐type mice, displaying potential as an amyloid burden marker of AD brains.

## Graphical abstract

Blood amyloid‐β (Aβ) can become a reliable surrogate biomarker if the issue of low concentration can be overcome. The brain‐to‐blood efflux driven by the oral administration of an Aβ disaggregator dissolves plaques and subsequently increases the plasma Aβ levels. This result reflects the brain amyloid burden and distinguishes Alzheimer transgenic mice and non‐Alzheimer mice.

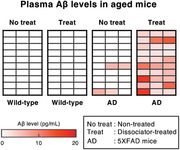

## 1 Introduction

Deposition of amyloid‐β (Aβ) is a key sign of Alzheimer's disease (AD).^[^ ^1^ ^]^ In the brain of AD patients, Aβ monomers are released from amyloid precursor protein by abnormal enzymatic cleavages and aggregate into neurotoxic soluble oligomers and insoluble plaques, which are the pathological processes leading to neurodegeneration. The alteration of Aβ levels in AD patients increased in the brain while concomitantly decreased in the cerebrospinal fluid (CSF) as the disease progresses, and Aβ can be detected by positron emission tomography (PET)^[^ ^2^ ^]^ and CSF tests.^[^ ^3^ ^]^ As Aβ deposition precedes the onset of AD symptoms by at least a decade,^[^ ^4^ ^]^ earlier diagnostic approaches, such as Aβ blood tests, are actively investigated, to screen at‐risk subjects and patients in asymptomatic stages prior to radiotracer injection and lumbar puncture.^[^ ^3^, ^5^ ^]^ However, Aβ blood tests suffer from two issues impeding the measurement and data interpretation: low concentration of Aβ in blood and unclear brain‐blood correlation of Aβ. First, blood Aβ and its AD‐related perturbation are found at pg mL^−1^ level, which is below the limit of detection (LOD) of conventional Aβ‐targeting enzyme‐linked immunosorbent assay (ELISA) kits. Secondly, it is unclear whether blood Aβ, and its concentration alterations during AD development, is mainly derived from the brain, the peripheral nervous systems, or both yet. Consequently, the manner in which blood Aβ concentration alters in AD patients and cognitively normal subjects vary depending on study sites; overall longitudinal changes of blood Aβ does not correlate with the increasing pattern of brain Aβ deposition nor the decreasing behavior of Aβ in CSF.

We hypothesized that chemical‐driven dissociation of Aβ aggregates in AD brains would release a substantial portion of soluble, smaller Aβ species penetrating the blood‐brain barrier and directly induce an increase of the blood Aβ level. Thus, an in vitro diagnostic approach measuring plasma A*β* was developed by incorporating anti‐Aβ42 ELISA and oral administration of a plaque‐dissociating chemical agent. The hypothetical concept was tested in the preclinical level of 5XFAD transgenic mouse model (B6SJL‐Tg(APPSwFlLon, PSEN1*M146L*L286V) 6799Vas/Mmjax, *n* = 93) and wild‐type mice (B6SJL, *n* = 25) utilizing 4‐(2‐hydroxyethyl)‐1‐piperazinepropanesulphonic acid (EPPS) as a chemical dissociator of brain plaques.^[^ ^6^ ^]^ Given that 5XFAD mice begins Aβ plaque deposition in the brain as early as two months of age,^[^ ^7^ ^]^ we prepared three age groups, young‐adult (2‐ to 3‐month‐old), adult (6‐ to 7‐month‐old), and aged (11‐ to 12‐month‐old), of both male and female mice, in addition to aged (11‐ to 12‐month‐old) wild‐type groups as non‐AD controls. The goal of our study was to examine 1) whether the plaque dissociation derives an increase of blood Aβ level that is within the measurable detection range of ELISA, 2) whether the chemical‐driven outflow of Aβ to blood reflects the amyloid burden in the brain, and 3) whether the measurement of plasma Aβ levels after amyloid dissociator treatment can distinguish AD and non‐AD mice.

## 2 Results

### 2.1 Increased Plasma Aβ Levels in 5XFAD Mice by the Administration of an Aβ Aggregates Dissociator

**Figure 1:**
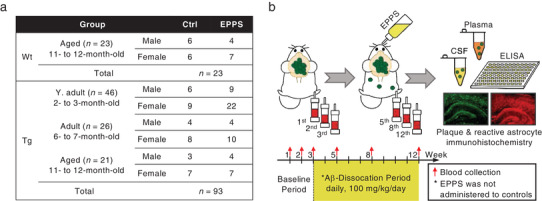
Experimental scheme of the entire study. a) Table listing 16 groups for blood collections. Wild‐type male (Aged, *n* = 10) and female (Aged, *n* = 13). Transgenic male (Y. adult, *n* = 15; Adult, *n* = 8; Aged, *n* = 7) and female (Y. adult, *n* = 31; Adult, *n* = 18; Aged, *n* = 14). b) Schematic overview of blood collections with an Aβ aggregates dissociator (EPPS) administration for analyses of Aβ in plasma, CSF, and brain. Wt, Wild‐type; Tg, Transgenic; Ctrl, Control, Y. adult, Young‐adult.

5XFAD transgenic (*n* = 93) mice were distributed by gender and in three age groups: young‐adult (2‐ to 3‐month‐old, *n* = 46), adult (6‐ to 7‐month‐old, *n* = 26), and aged (11‐ to 12‐month‐old, *n* = 21). The aged wild‐type (*n* = 23) mice of both male and female were also prepared as non‐AD controls (**Figure** **1**a). A total of 56 transgenic and 11 wild‐type mice received ad libitum oral administration of 100 mg kg^−1^ day^−1^ EPPS in drinking water, while 37 transgenic and 12 wild‐type mice received no additional material, for nine weeks. The 12‐week experiment comprises 1) a three‐week baseline period to identify the basal plasma Aβ levels and 2) an Aβ‐dissociation period to observe the alteration of plasma Aβ levels by an Aβ dissociator treatment for additional nine weeks (Figure 1b). Blood was collected at 1st, 2nd, and 3rd weeks of the baseline period and at 5th, 8th, and 12th weeks of the Aβ‐dissociation period from the lateral saphenous vein of each mouse to obtain minimal blood sampling (6 µL g^−1^).^[^ ^8^ ^]^ Subsequently, we performed histochemical analyses to observe plaques and reactive astrocytes by ex vivo brain staining and measured plasma and CSF Aβ levels by ELISA (Figure 1b). All blood and CSF samples were mixed immediately with a protease inhibitor cocktail to prevent the degradation of Aβ and diluted in a 5‐fold and 60‐fold, respectively, for quantitative analyses.

**Figure 2:**
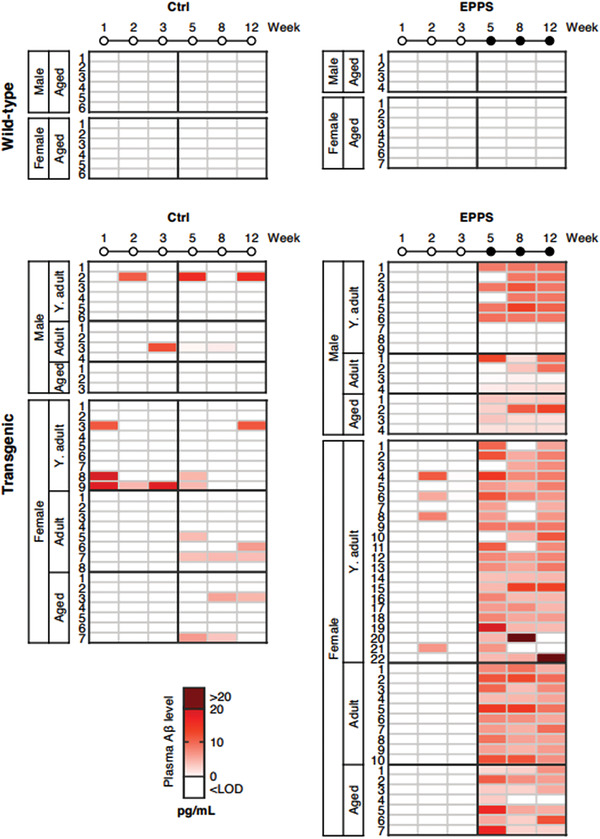
Alterations of plasma Aβ levels in 5XFAD mice by the administration of an Aβ aggregates dissociator. Heatmap of plasma Aβ levels (white to red, 0 to 20 pg mL^−1^) in six blood samples of individuals in all groups of wild‐type and transgenic mice (Water, ○; EPPS, ●). Wild‐type male (Aged, *n* = 10) and female (Aged, *n* = 13). Transgenic male (Y. adult, *n* = 15; Adult, *n* = 8; Aged, *n* = 7) and female (Y. adult, *n* = 31; Adult, *n* = 18; Aged, *n* = 14). Y. adult, Young‐adult; CSF, Cerebrospinal fluid; Ctrl, Control; LOD, Limit of detection.

The plasma Aβ levels of all mice were displayed using a color‐coded heatmap based on the level from 0 to 20 pg mL^−1^ (white to red) and the white color comprises values below the LOD of anti‐Aβ42 ELISA kits (**Figure** **2**; Figure S1a,b, Supporting Information). As expected, all wild‐type mice exhibited negative values of plasma Aβ in all the collected blood samples. Similarly, the plasma Aβ levels of transgenic control mice were predominantly detected as negative values (white), regardless of age, indicating that measurement of plasma Aβ level is challenging to distinguish between wild‐type and transgenic mice without any supplementary treatment. However, in transgenic mice treated with EPPS, we found a substantial increase of plasma Aβ concentration as indicated by the contrasting color change of white to red from the baseline period to the Aβ‐dissociation period (Figure 2). The distinctive data support our hypothesis that the administration of EPPS disaggregates Aβ plaques and a consequent brain‐to‐blood efflux of Aβ may occur, increasing the levels of plasma Aβ.

### 2.2 Alteration of Plasma Aβ Levels by the Administration of an Aβ Aggregates Dissociator

**Figure 3:**
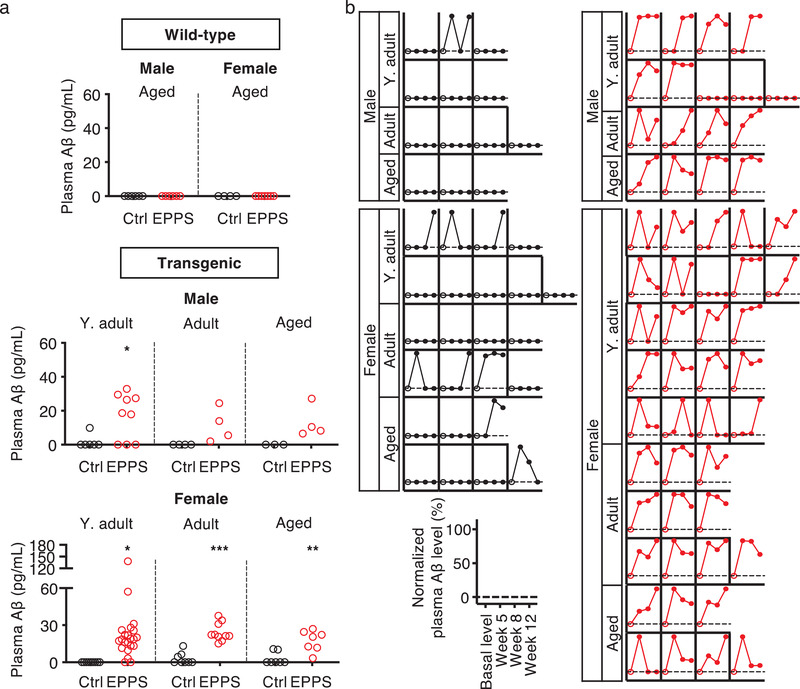
Cumulating and tracking the altered plasma Aβ levels in 5XFAD mice after the administration of an Aβ aggregates dissociator. a) Cumulative plasma Aβ values of blood samples collected at weeks 5, 8, and 12 from both wild‐type and transgenic mice groups (Control, black; EPPS, red). Wild‐type male (Aged, *n* = 10) and female (Aged, *n* = 13). Transgenic male (Y. adult, *n* = 15; Adult, *n* = 8; Aged, *n* = 7) and female (Y. adult, *n* = 31; Adult, *n* = 18; Aged, *n* = 14). b) Individually normalized plasma Aβ tracking data of blood samples collected at week 5, 8, and 12 for each mouse in all groups using a basal plasma Aβ and maximal plasma Aβ levels of individuals (Individual basal plasma Aβ level, open circle; closed circle: EPPS treatment; Control, black; EPPS, red). Y. adult, Young‐adult; CSF, Cerebrospinal fluid; Ctrl, Control; LOD, Limit of detection. The error bars represent the SEM. All statistical analyses were performed by two‐tailed unpaired *t*‐test with the comparison to each control mice of groups (*P<0.05, **P<0.01, ***P<0.001; other comparisons were not significant). Scale bar = 750 µm.

As orally administered EPPS increased the plasma Aβ levels, we further compared the plasma Aβ alteration between control and EPPS‐treated transgenic mice. The negative values were zeroed and the averaged basal plasma Aβ levels of each mouse were deducted from the total altered plasma Aβ levels of individuals, which were obtained by cumulating the three plasma Aβ levels of the Aβ‐dissociation period. As expected, the cumulated plasma Aβ levels of individuals were mostly zero in all ages and genders of wild‐type and control groups, while considerable amounts of plasma Aβ were observed in EPPS‐treated mice (**Figure** **3**a).

In addition, we observed an age‐dependent decrease of plasma Aβ levels in EPPS‐treated mice for both genders. To monitor the plasma Aβ alteration of each mouse, all plasma Aβ levels of the control and EPPS‐treated transgenic groups were tracked by normalizing the basal Aβ level (0%) and maximum plasma Aβ level (100%) of individuals (Figure 3b). Resultantly, substantial levels of plasma Aβ altered in the EPPS‐administered mice (red) compared to the control mice (black). Although both groups of mice exhibit increased plasma Aβ levels, greater amounts of EPPS‐treated mice than the control suggests that EPPS treatment contributes to the elevation of the total plasma Aβ levels.

### 2.3 The Elevation of Aβ Levels in Blood Derived by the Chemical‐Driven Efflux of Brain Aβ after the Treatment of an Aβ Aggregates Dissociator

**Figure 4:**
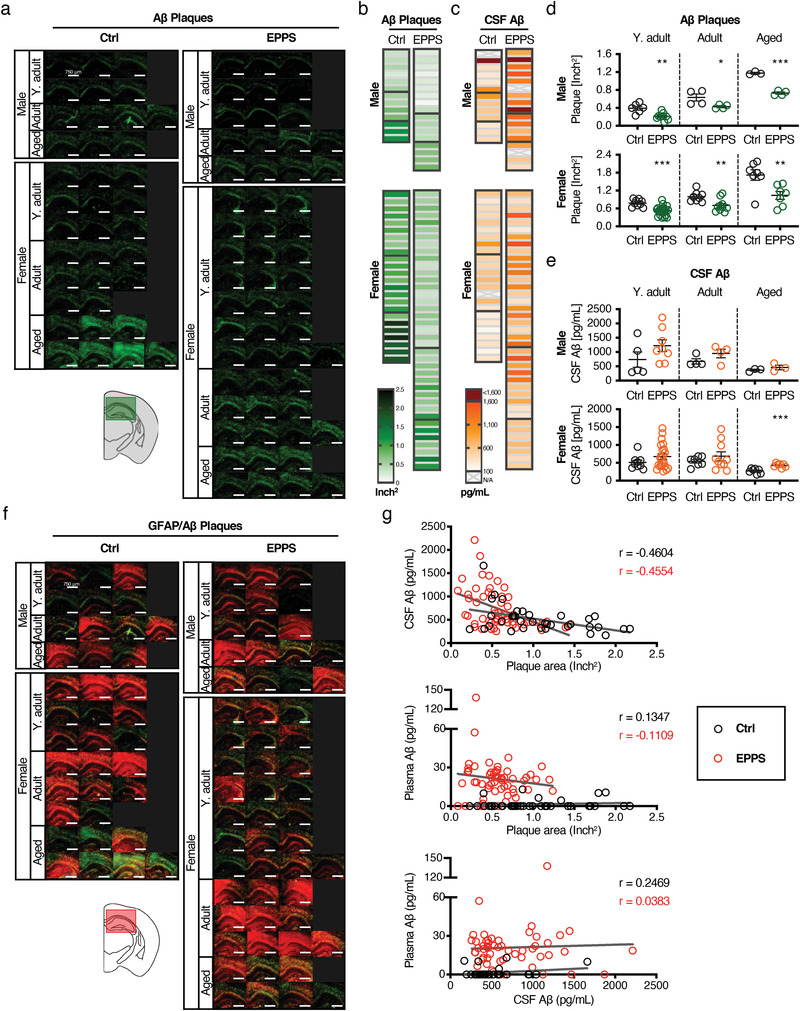
Aβ levels in the brain and CSF of 5XFAD mice with the administration of an Aβ aggregates dissociator. a) ThS stained Aβ plaques in the brain tissue of each mouse. In a brain atlas, a green box indicates an area for brain images and gray color indicates the region of quantification of total Aβ plaque area. b,c) Heatmap representing (b) the total Aβ plaque area (inch^2^) and (c) CSF Aβ (pg mL^−1^) of each mouse. d,e) Comparison data of (d) Aβ plaque area and (e) CSF Aβ of each group with the control. The crossed‐out data in the CSF heatmap indicates a sample loss. (Control, black; Aβ plaque area in EPPS administration, green; CSF Aβ in EPPS administration, orange). f) The merged brain images of reactive astrocytes immunostaining (GFAP, red) with ThS stained A*β* plaques (GFAP/Aβ plaques). The red box of brain atlas image indicates an area for brain images. g) Analyses of correlations between the levels of CSF Aβ and Aβ plaques (top), plasma Aβ and Aβ plaques (middle), and plasma and CSF Aβ (bottom) (Control, black; EPPS, red). The sample size is as follows: Transgenic male (Y. adult, *n* = 15; Adult, *n* = 8; Aged, *n* = 7) and female (Y. adult, *n* = 31; Adult, *n* = 18; Aged, *n* = 14). Y. adult, Young‐adult; CSF, Cerebrospinal fluid; Ctrl, Control. The error bars represent the SEM and the statistical analyses were performed by two‐tailed unpaired *t*‐test with the comparison to the age‐matched control group (*P < 0.05, **P < 0.01, ***P < 0.001; other comparisons were not significant). Scale bar = 750 µm.

To investigate whether the increased plasma Aβ upon administration of EPPS was derived from the brain, we analyzed changes in cerebral plaque load (thioflavin S, ThS), reactive astrocyte levels (anti‐glial fibrillary acidic protein, anti‐GFAP), and CSF Aβ concentrations (anti‐Aβ42 ELISA) (**Figure** **4**). Each hemisphere image represents an individual mouse as shown in Figure 4a, and the total plaque area was presented by a heatmap in color codes from white to green for 0 to 2.4 inch^2^ (Figure 4b). Plaque burden increased age‐dependently in both the control (black) and EPPS‐treated (green) groups. However, EPPS treatment significantly reduced plaques compared to the age‐matched controls (Figure 4b,d). The values for areas and numbers of total plaques are provided in Figure S2, Supporting Information, including the regional analyses of plaques in hippocampus and cortex. The representative wild‐type mice as non‐AD controls were displayed in Figure S3a, Supporting Information. The CSF Aβ levels were additionally evaluated using a heatmap ranging from 100 to 1,600 pg mL^−1^ (white to orange color‐coded) (Figure 4c). As CSF Aβ levels reversely reflect the amount of Aβ plaques in the brain,^[^ ^1^, ^9^ ^]^ we found an age‐dependent decrease of CSF Aβ levels in both the control and EPPS‐treated groups. However, EPPS‐treated mice displayed higher levels of CSF Aβ compared to the control, indicating that solubilized brain Aβ by EPPS was transported to CSF (Figure 4e). Interestingly, CSF Aβ levels of young mice were distributed in a varied range. A similar trend was also observed in the basal plasma Aβ levels (Figure 2) within the young age group. The early and aggressive Aβ accumulation in 5XFAD mouse model could be considered as the feature influencing the variation of Aβ levels in biofluids, such as CSF and plasma.

Besides the reduction of Aβ plaques, we also identified diminished plaque‐associated reactive astrocytes, visualized via co‐staining of GFAP with Aβ plaques (merged images) (Figure 4f; Figure S3b, Supporting Information). We then further investigated the correlation among the Aβ levels in blood, CSF, and brain by the administration of an Aβ aggregates dissociator (Figure 4g). An inverse correlation was observed, displaying the decrease of CSF Aβ while Aβ deposits increased in both the control and EPPS‐treated mice.

Comparatively, the EPPS‐treated group exhibited higher levels of CSF Aβ and reduced amounts of plaques than the control. Between the levels of plasma Aβ and plaques or CSF Aβ, correlations were not observed in the control nor the EPPS‐treated group, despite the age‐dependent alteration of plasma Aβ in AD mice with EPPS administration.

## 3 Discussion

We focused on the blood diagnosis of AD to measure brain Aβ in blood without invasive assessment for Aβ measurement. As individual variations cause difficulty determining the cut‐off value of plasma Aβ concentration, we identified and utilized the basal plasma Aβ level of each subject as a self‐standard to track the alteration of plasma Aβ concentration.

In this study, the long‐term administration of EPPS not only elevated plasma Aβ levels, but reduced Aβ plaques, allowing us to predict the presence of Aβ plaques in the brain without PET imaging or CSF collections for Aβ detection. Thus, this can be applied to distinguish between AD and non‐AD by measuring Aβ in blood samples obtained from pre‐ and post‐administration of an Aβ aggregates dissociator.

Since we obtained quantification of Aβ plaques by ex vivo staining, this study lacks to assess chronological images of the brain for each mouse. When a database of the plasma Aβ levels and their correspondent PET imaging obtained from AD patients is developed, the trend of plasma Aβ level changes may display a skewed bell‐shaped curve that escalates along the presence of Aβ plaques and declines as plaques decrease in the brain. The database can be utilized to further facilitate the quantification of plaques by measuring the plasma Aβ without brain imaging.

Recently, the Food and Drug Administration has approved aducanumab (Aduhelm) as the first medication for AD that triggers Aβ clearance in the brain.^[^ ^10^ ^]^ The approval of the disease‐modifying drug for AD can support in assenting other drug candidates with a similar mode of action. For the development and validation of the drugs targeting Aβ clearance,^[^ ^11^ ^]^ it is necessary to analyze their efficacy on brain Aβ reduction. Our approach using blood tests for identification and diminution of Aβ plaques in the brain can be applied as a powerful tactic to diagnose and treat AD.

## 4 Experimental Section

### Animal Study

Male and female 5XFAD transgenic mice (B6SJL‐Tg(APPSwFlLon, PSEN1*M146L*L286V) 6799Vas/Mmjax) and WT (B6SJL) mice were obtained from Jackson laboratory (USA). All mice were maintained in groups of 4–5/cage in the animal facility of Yonsei University (Seoul, Korea) and bred under controlled temperature and humidity given with 12:12 hour light‐dark cycle and access to food and water ad libitum. Depending on the availability of the experimental animals, the number of mice varied to be used for each group. All animal experiments were carried out in accordance with the National Institutes of Health Guide for the Care and Use of Laboratory Animals, and the Animal Institutional Animal Care and Use Committee of Yonsei University (Seoul, Korea).

### Drug Administration

An Aβ plaque disaggregating molecule, 4‐(2‐hydroxyethyl)‐1‐piperazinepropanesulphonic acid (EPPS) was prepared as an Aβ aggregates dissociator.^[^ ^6^ ^]^ Transgenic mice in young‐adult (2‐ to 3‐month‐old, *n* = 46), adult (6‐ to 7‐month‐old, *n* = 26), and aged (11‐ to 12‐month‐old, *n* = 21) group in addition to aged wild‐type mice group (11‐ to 12‐month‐old, *n* = 25), of both male and female, were given water ad libitum and weekly blood collection for three weeks. Afterwards, 56 mice (EPPS group) and 13 mice (wild‐type) received oral administration of 100 mg kg^−1^ day^−1^ EPPS, ad libitum, for nine weeks and 37 mice (control group) along with 12 mice (wild‐type) received no additional material. The duration of the experiment was 12 weeks, consisting of three weeks of the baseline period, given only water to all mice, and nine additional weeks of the Aβ‐dissociation period. Three blood collections of all mice occurred during the Aβ‐dissociation period at 5th, 8th, and 12th weeks. The water administered to all mice was freshly changed every other day. The amount of EPPS in drinking water was re‐calculated regularly to make 100 mg kg^−1^ day^−1^ by measuring the water intake for each cage and weekly evaluating the change in body weight of each mouse.^[^ ^12^ ^]^ After 12 weeks, all mice groups were sacrificed followed by extracting brain tissue samples and collecting CSF for Aβ measurement.

### Plasma Collection

For a repeat collection of blood, a minimum of 6 µL g^−1^ of blood was collected from the lateral saphenous vein to perform a quick and relatively less stressful method for animals without anesthesia using a tail vein restrainer (JeungDo Bio).^[^ ^8^ ^]^ For a clear view of the saphenous vein, a shaver (Philips) was used to remove the fur on the mice's thigh. While gently holding the hind leg of the mice, a lancet (23G, Greenlan) was pricked at the vein to collect the blood into the EDTA vacutainer tubes (Sarstedt) and gently inverted to mix then stored in ice. Blood samples were centrifuged for three minutes at 3,000 rpm in 4 °C. The plasma above the buffy coat was acquired and aliquoted into the 1.5 mL tubes followed by adding the protease inhibitor cocktail (cOmplete, Mini, Roche) into the plasma fraction and stored at −80 °C until analyses.

### Cerebrospinal Fluid Collection

The mice were sacrificed with 4% avertin (40 µg g^−1^, intraperitoneally) and the CSF samples were collected by puncturing into the cisterna magna of the mice with the hand‐made capillary. The protease inhibitor cocktail was added into the collected samples and stored at −80 °C until further analyses. After CSF collection, the animal was perfused with 0.9% saline and the excised half brains were fixed overnight in ice‐cold 4% paraformaldehyde at 4 °C and then immersed in 30% sucrose for 48 hours. The hemisphere was cut into 35 µm sections using a cryostat at −20 °C (Leica CM1860) to mount them onto the glass slides (Thermo Fisher Scientific).

### Immunohistochemistry of Brain Sections

The brain sections on the glass slides were washed with a phosphate‐buffered saline (PBS) solution. For antigen retrieval, slides were soaked in 1% sodium dodecyl solution diluted with PBS for 10 minutes and blocked with 20% horse serum albumin (HSA) in PBS for one hour at room temperature to prevent non‐specific binding.

Primary antibody of GFAP (Millipore, 1:300 in 5% HSA in PBS) was then treated at 4 °C for overnight and the fluorescence secondary antibody, goat anti‐chicken antibody conjugated with Alexa Fluor 568 (Life Technologies, 1:200 in PBS), was treated for one hour at room temperature. All steps after the secondary antibody treatment were performed in the dark. To visualize Aβ plaques, the tissues were stained with ThS solution (Sigma‐Aldrich) dissolved in 50% ethanol at 500 µm for seven minutes in the dark followed by washing the slides at 100, 90, and 70% ethanol solutions for one minute each before transferring into PBS. Tissues images were obtained by a fluorescence microscope (Leica DM2500) with a LAS X software program. Aβ plaques were analyzed by Image‐J software.

### Aβ42 Enzyme‐Linked Immunosorbent Assay (ELISA) of Mice Samples

To detect and quantify the levels of human Aβ42 in plasma and CSF, ELISA was conducted using a human Aβ42 ultrasensitive ELISA kit (Invitrogen) and performed according to the protocol. Briefly, plasma and CSF samples were prepared by diluting up to 1:5 or 1:60 fold, respectively, in a standard diluent buffer with a protease inhibitor cocktail. The human Aβ42 standard was prepared in serial dilutions as follows: 100, 50, 25, 12.5, 6.25, 3.13, 1.56, and 0 pg mL^−1^. The standards and the mice samples were then added to the appropriate wells of the plate. The human Aβ42 detection antibody was then inserted to wells and the detection antibody was treated for overnight at 4 °C. After the plate was washed using a wash buffer, secondary antibody (anti‐rabbit IgG horseradish peroxidase) was added and incubated for 45 minutes at room temperature. After washing the wells, a chromogen solution was treated to the plate for 30 minutes, in the dark, and added a stop solution followed by measurement at 450 nm using SpectraMax M2 microplate reader (Molecular Devices).

### Statistical Analyses

Statistical analyses were conducted with GraphPad Prism 9. All analyses used two‐tailed unpaired *t*‐tests (*P < 0.05, **P < 0.01, ***P < 0.001, other comparisons not significant). The error bars represent the SEM.

## Conflict of Interest

H.V.K., H.Y.K., and Y.K. are inventors on patents related to this work issued by Korea Institute of Science and Technology (KIST, no. US10006920, registered on 26 June 2018 and no. KR1016192380000, registered on 2 May 2016). The authors declare that they have no other competing interests.

## Author Contributions

D.L., H.V.K., H.Y.K., and Y.K. conceived the study; D.L. performed the experiments; D.L., H.Y.K., and Y.K. analyzed the data and wrote the manuscript; D.L., H.Y.K., and Y.K. revised the manuscript; and H.Y.K. and Y.K. supervised the project.

## Supporting information

Supporting Information

## Data Availability

The data that support the findings of this study are available from the corresponding author upon reasonable request.
