# Supplementary material for: Chemical‐Driven Outflow of Dissociated Amyloid Burden from Brain to Blood
Source: Adv Sci (Weinh). 2022 Feb 2;9(12):2104542. doi: 10.1002/advs.202104542 (PMC9036038; doi:10.1002/advs.202104542)
Supplement: Supplementary file 1 — Supporting Information [file ADVS-9-2104542-s001.pdf]

ADVANCED SCIENCE
Open Access

Supporting Information

for *Adv. Sci.*, DOI 10.1002/advs.202104542

Chemical-Driven Outflow of Dissociated Amyloid Burden from Brain to Blood

Donghee Lee, Hyunjin Vincent Kim, Hye Yun Kim and YoungSoo Kim**

ADVANCED SCIENCE
Open Access

Supporting Information

for *Adv. Sci.*, DOI: 10.1002/advs.202104542

Chemical-driven outflow of dissociated amyloid burden from brain to blood

Donghee Lee, Hyunjin Vincent Kim, Hye Yun Kim, and YoungSoo Kim**

Supporting Information

Chemical-driven outflow of dissociated amyloid burden from brain to blood

Donghee Lee, Hyunjin Vincent Kim, Hye Yun Kim, and YoungSoo Kim**

D. Lee, Dr. H. Y. Kim, Prof. Y. Kim
Department of Pharmacy, College of Pharmacy
Yonsei University
85 Songdogwahak-ro, Yeonsu-gu, Incheon 21983, South Korea
E-mail: y.kim@yonsei.ac.kr; hyeyunkim@yonsei.ac.kr

D. Lee, Dr. H. Y. Kim, Prof. Y. Kim
Yonsei Institute of Pharmaceutical Sciences, College of Pharmacy
Yonsei University
85 Songdogwahak-ro, Yeonsu-gu, Incheon 21983, South Korea

H. V. Kim
Korea Institute of Science and Technology (KIST), University of Science and Technology (UST)
5, Hwarang-ro 14-gil, Seongbuk-gu, Seoul 02792, South Korea

Prof. Y. Kim
Department of Integrative Biotechnology and Translational Medicine
Yonsei University
85 Songdogwahak-ro, Yeonsu-gu, Incheon 21983, South Korea

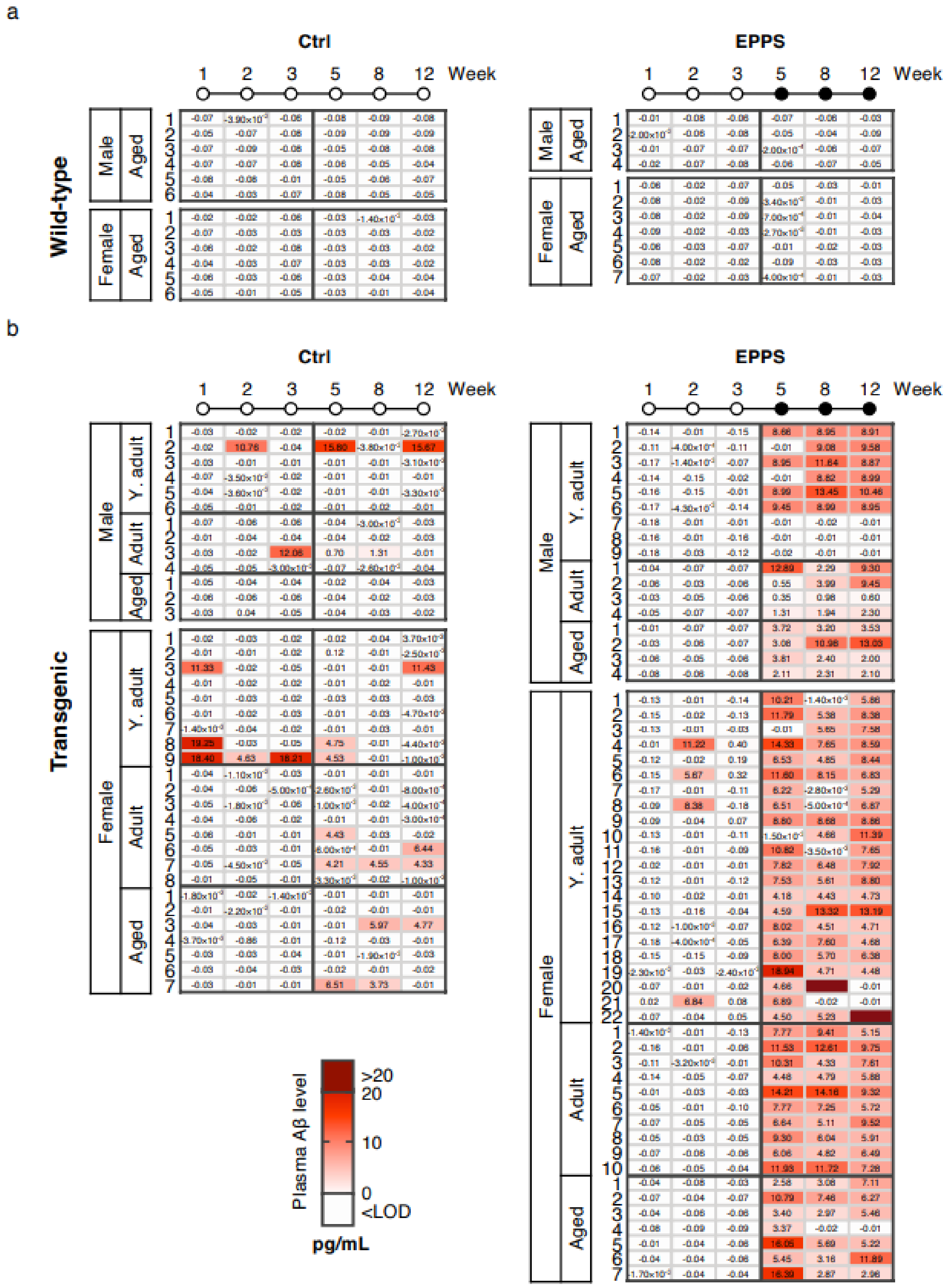

Figure S1. Alterations of plasma Aβ levels in 5XFAD mice by the administration of an Aβ aggregates dissociator, EPPS. a,b) Heatmap of plasma Aβ levels with the raw data (white to red, 0 to 20 pg mL^{-1}) in six blood samples of individuals in all groups of wild-type and transgenic mice (Water, ○; EPPS, ●). Wild-type male (Aged, *n* = 10) and female (Aged, *n* =

13). Transgenic male (Y. adult, n = 15; Adult, n = 8; Aged, n = 7) and female (Y. adult, n = 31; Adult, n = 18; Aged, n = 14). Y. adult, Young-adult; CSF, Cerebrospinal fluid; Ctrl, Control; LOD, Limit of detection.

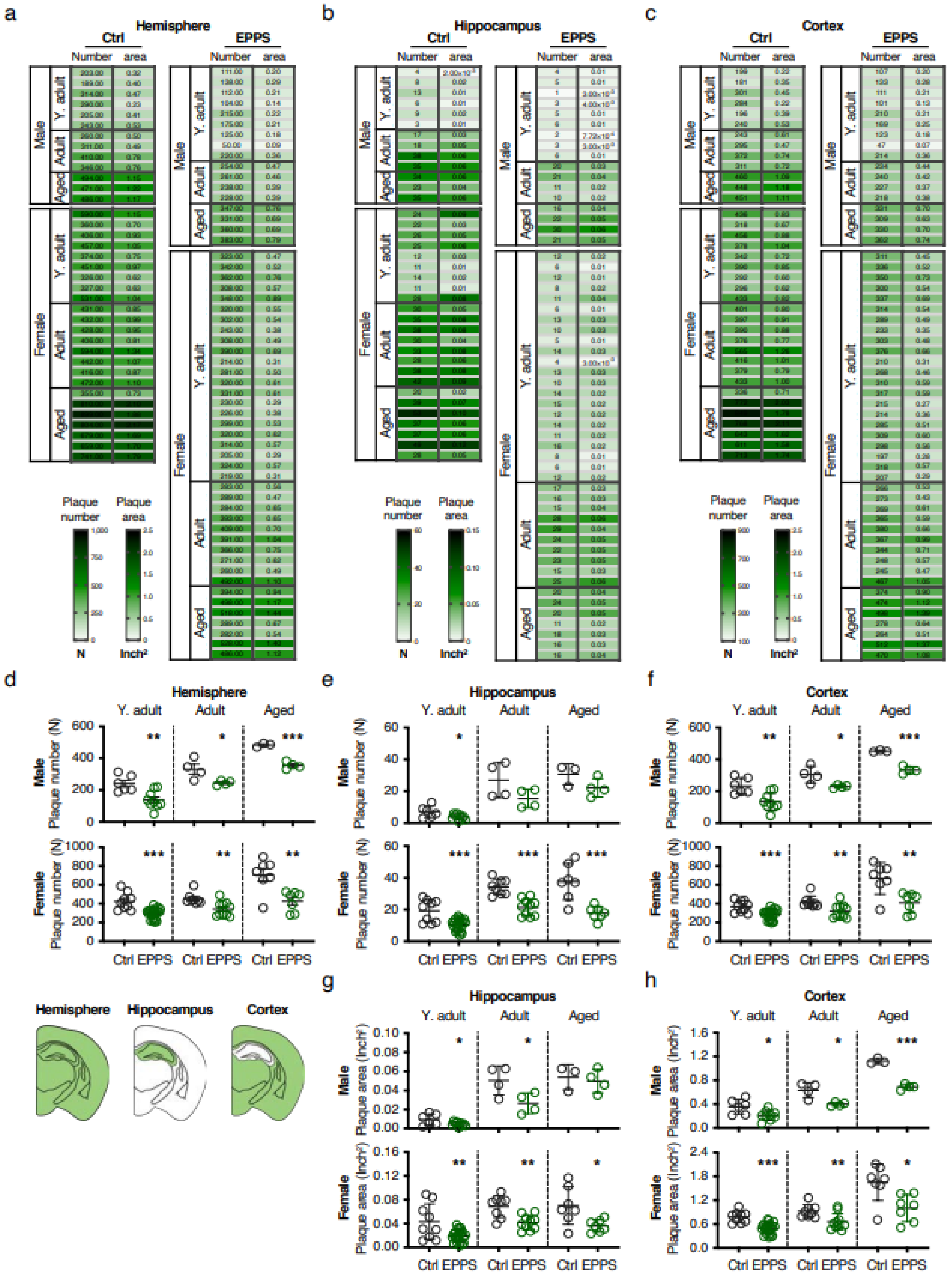

Figure S2. Aβ levels in hippocampus, cortex, and hemisphere in 5XFAD mice by administration of Aβ aggregates dissociator, EPPS. a–c) Heatmap of total Aβ plaque number (N) and area ($Inch^2$) with the raw data of each mouse in a) hemisphere, b) hippocampus, and c) cortex. d–h) Comparison data of d–f) Aβ plaque number and g,h) area of each group with control. (Control, ○; Plaque area in EPPS administration, ○). The green color of brain atlas images indicates an area for the regional plaque quantification. Transgenic male (Y. adult, n = 15; Adult, n = 8; Aged, n = 7) and female (Y. adult, n = 31; Adult, n = 18; Aged, n = 14). Y. adult, Young-adult; CSF, Cerebrospinal fluid; Ctrl, Control. The error bars represent the SEM, and all statistical analyses were performed by two-tailed unpaired t-test with the comparison to age-matched control groups (*P < 0.05, **P < 0.01, ***P < 0.001; other comparisons were not significant). Scale bar = 750 μm.

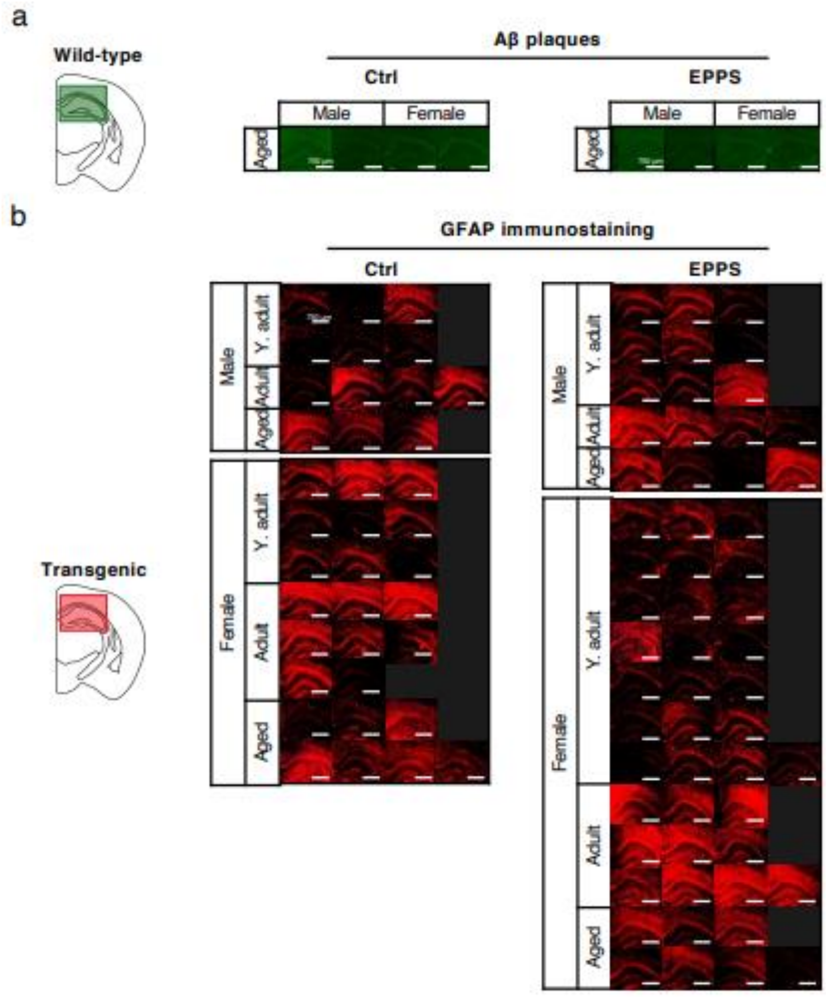

Figure S3. Reactive astrocytes and Aβ plaques in the brain of 5XFAD mice with the administration of an Aβ aggregates dissociator, EPPS. a) Representative brain images of wild-type by ThS staining of Aβ plaques. The green box of brain atlas image indicates an area for

the brain image. b) The brain images of each group with reactive astrocytes immunostaining (GFAP). The red box of brain atlas image indicates an area for the brain image. Wild-type male (Aged, n = 4) and female (Aged, n = 4). Transgenic male (Y. adult, n = 15; Adult, n = 8; Aged, n = 7) and female (Y. adult, n = 31; Adult, n = 18; Aged, n = 14). Y. adult, Young-adult; Ctrl, Control; ThS, Thioflavin S; GFAP, Glial fibrillary acidic protein. Scale bar = 750 μm.
